# Risk Factors Associated With Mortality Among Residents With Coronavirus Disease 2019 (COVID-19) in Long-term Care Facilities in Ontario, Canada

**DOI:** 10.1001/jamanetworkopen.2020.15957

**Published:** 2020-07-22

**Authors:** David N. Fisman, Isaac Bogoch, Lauren Lapointe-Shaw, Janine McCready, Ashleigh R. Tuite

**Affiliations:** 1 Dalla Lana School of Public Health, University of Toronto, Toronto, Ontario, Canada; 2 Department of Medicine, University of Toronto, Toronto, Ontario, Canada; 3 Division of General Internal Medicine and Geriatrics, University Health Network, Toronto, Ontario, Canada; 4 Division of Infectious Diseases, University Health Network, Toronto, Ontario, Canada; 5 Department of Medicine and Division of Infectious Diseases, Michael Garron Hospital, Toronto, Ontario, Canada

## Abstract

**Importance:**

The coronavirus disease 2019 (COVID-19) pandemic has been particularly severe among individuals residing in long-term care (LTC) facilities. As of April 10, 2020, half of Canada’s COVID-19 deaths had occurred in LTC facilities.

**Objective:**

To better understand trends and risk factors associated with COVID-19 death in LTC facilities in Ontario, Canada.

**Design, Setting, and Participants:**

This cohort study of 627 LTC facilities included 269 total individuals who died of COVID-19 in Ontario to April 11, 2020, and 83 individuals who died of COVID-19 in Ontario LTC facilities to April 7, 2020. Because population denominators were not available for LTC residents, they were approximated as the total number of LTC facility beds in Ontario (79 498), assuming complete occupancy.

**Exposures:**

Confirmed or suspected COVID-19 outbreaks; confirmed COVID-19 infection among residents and staff, diagnosed by real-time polymerase chain reaction testing.

**Main Outcomes and Measures:**

COVID-19–specific mortality incidence rate ratios (IRRs) for LTC residents were calculated with community-living Ontarians older than 69 years as the comparator group. Count-based regression methods were used to model temporal trends and to identify associations of infection risk among staff and residents with subsequent LTC resident death. Model-derived IRRs for COVID-19–specific mortality were generated through bootstrap resampling (1000 replicates) to generate median and 95% credible intervals for IRR over time.

**Results:**

Of 627 LTC facilities, 272 (43.4%) reported COVID-19 infection in residents or staff. Of 1 731 315 total individuals older than 69 years living in Ontario during the study period, 229 (<0.1%) died; of 79 498 potential residents in LTC facilities, 83 (0.1%) died. The IRR for COVID-19–related death in LTC residents was 13.1 (95% CI, 9.9-17.3) compared with community-living adults older than 69 years. The IRR increased sharply over time and was 87.3 (95% credible interval, 6.4-769.8) by April 11, 2020. Infection among LTC staff was associated with death among residents with a 6-day lag (eg, adjusted IRR for death per infected staff member, 1.17; 95% CI, 1.11-1.26).

**Conclusions and Relevance:**

In this cohort study of COVID-19–related deaths during the pandemic in Ontario, Canada, mortality risk was concentrated in LTC residents and increased during a short period. Early identification of risk requires a focus on testing, providing personal protective equipment to staff, and restructuring the LTC workforce to prevent the movement of COVID-19 between facilities.

## Key Points

**Question:**

How does the risk of death from coronavirus disease 2019 (COVID-19) among residents of long-term care (LTC) homes compare with that among the general population?

**Findings:**

In this cohort study of 627 LTC facilities, the incidence rate ratio for COVID-19–related death among LTC residents was 13 times higher than that among community-living adults older than 69 years.

**Meaning:**

In this study, the risk of COVID-19–related death was elevated among LTC residents, highlighting the need for improved infection control, widespread testing, access to personal protective equipment, and other supports to protect this vulnerable population.

## Teaser

This cohort study examines the trends and risk factors associated with coronavirus disease 2019 (COVID-19) death in long-term care facilities in Ontario, Canada.

## Introduction

Communicable diseases do not respect boundaries, but they also do not affect all members of our society equally. Since the initial recognition of severe acute respiratory syndrome coronavirus 2 (SARS-CoV-2) in January 2020, it has become starkly apparent that the virus disproportionately affects older individuals and those with more comorbidities, among whom mortality is highest.^1^ In Canada, we had an early indication of the particular susceptibility of those living in long-term care (LTC) facilities. The first confirmed death in the country, which occurred on March 9, 2020, was a resident of a North Vancouver LTC home.^2^

Since then, outbreaks in LTC facilities have been identified across Canada. As of April 9, 2020, nearly 50% (198 of 401 [49.4%]) of all deaths attributable to COVID-19 in the country have occurred among LTC residents.^3^ In addition to the age and comorbidity profiles of residents, other factors make LTC facilities especially susceptible to infectious disease outbreaks.^4^ These include a lack of access to testing and personal protective equipment, the close quarters of residents, the difficulty of maintaining physical distancing among mobile patients with dementia, and a precariously employed workforce that can transmit the virus across LTC sites.^5,6,7^

Recognizing and quantifying the scope of the issue and identifying which resident and worker factors may contribute to sudden increases in deaths is an important first step toward ensuring that comprehensive policies and response measures are in place to protect residents and the health care workers who provide essential services to them. We sought to evaluate the risk of death among residents of LTC facilities and to identify risk factors associated with mortality using data from the province of Ontario.

## Methods

Data for this study were obtained from the Ontario Ministry of Health and Long-term Care as part of the province’s emergency modeling table. The study was approved by the Research Ethics Board of the University of Toronto. The data were deidentified, and a waiver of informed consent was granted because the data were collected for public health surveillance purposes. The data included the following: (1) age and date of death of Ontario residents who tested positive for COVID-19 and (2) cumulative death and positive COVID-19 case counts by date among LTC residents and staff. Confirmed cases were diagnosed by real-time polymerase chain reaction testing.^8^ The available time series for LTC facilities covered the 10-day period from March 29 to April 7, 2020, inclusive; however, deaths before March 29 were included as part of the cumulative counts. The provincial death data included the period from March 3 to April 11, 2020. Daily deaths and cases were estimated by taking the difference of cumulative cases and deaths on sequential days.

Population denominators for deaths among non-LTC residents were derived from Statistics Canada estimates for the appropriate age groups. Population denominators were not available for LTC facilities and were approximated as the total number of facility beds in Ontario (ie, 79 498) assuming complete occupancy. Age data for deaths reported in LTC facilities were not available, but data show that approximately 93% of residents in LTC facilities are aged 65 years or older.^9^ Although the combination of aggregate and individual-level data made our cohort atypical, we have adhered as closely as possible to the Strengthening the Reporting of Observational Studies in Epidemiology (STROBE) reporting guideline for cohort studies.^10^

### Statistical Analysis

We compared differences in characteristics between LTC facilities that reported cases and those not reporting cases. Differences in proportions were tested by χ^2^ tests, and differences in bed numbers were tested with the Wilcoxon rank sum test (α = .05).

In our main analysis, we estimated incidence rate ratios (IRRs) for COVID-19 deaths in the LTC resident population compared with deaths in community-living adults in Ontario older than 69 years. In sensitivity analyses, we compared outcomes in LTC residents with COVID-19 deaths in the entire Ontario population, the population older than 59 years, and the population older than 79 years. Cumulative deaths at the end of the respective time series were considered, with risk in both LTC residents and non-LTC residents considered to have begun on March 3, 2020. However, person-time denominators were adjusted for the fact that the LTC facility database ended 4 days before the provincial death database (ie, 36 days in the LTC population and 40 days in the general Ontario population). Statistical significance was evaluated with a χ^2^ test (α = .05).

We also created a negative binomial regression model to evaluate the change in IRR among LTC residents compared with the non-LTC population from March 29 to April 7, 2020. Negative binomial regression was performed as a deviance statistic test and suggested that the data were not Poisson distributed. Non-LTC residents older than 69 years were used as the comparator population, and model offsets were the population denominators described previously. The model included 3 covariates, as follows: LTC residence, time (centered on April 3, 2020), and an interaction term of LTC residence (ie, yes/no) multiplied by time. Model estimations were generated both forward and backward to generate graphical representations of how risk in the LTC population has changed over time. Model-derived IRRs for COVID-19–specific mortality were generated through bootstrap resampling (1000 replicates) to generate medians and 95% credible intervals (CrIs) for IRRs over time.

Lastly, we created Poisson regression models that evaluated risk of death within LTC facilities as a function of the number of residents with laboratory-confirmed infection as well as staff with confirmed infection at lags of 0 to 7 days, adjusting for date. Given that our model could not accommodate multiple simultaneous lags, we constructed 8 models, each of which included cumulative infections among resident and staff at identical lags (0-7 days). Deviance statistics indicated that counts were Poisson distributed. We used the number of LTC beds as model offsets, and confidence intervals were adjusted for clustering by LTC facility. All analyses were conducted in Stata version 14 (StataCorp). The figures were made using ggplot in R version 1.2.1335 (R Project for Statistical Computing). Statistical significance was evaluated with 2-sided *z* scores set at α = .05.

## Results

A total of 627 LTC facilities were included in the provincial data set; of these, 272 (43.4%) were identified as having either confirmed or suspected COVID-19 infection in residents or staff. No significant differences between LTC facilities with and without confirmed COVID-19 infections were seen in number of licensed beds, operator type (eg, for profit vs nonprofit), or geographic location in Ontario (Table 1).

**Table 1.** Characteristics of Ontario Long-term Care Facilities Included in Database

Characteristic	All facilities, No. (%)	No./total No. (%)		*P* value^b^
		With COVID-19 cases^a^	Without COVID-19 cases^a^	
Facilities	627 (100)	272/627 (43.4)	355/627 (56.6)	NA
Total beds	79 498	36 441/79 498 (45.8)	43 057/79 498 (54.2)	NA
Beds, median (range)	120 (9-543)	118 (9-456)	120 (12-543)	.12
Region				
Central	123 (19.6)	62/123 (50.4)	61/123 (49.6)	.06
East	165 (26.3)	74/165 (44.8)	91/165 (55.2)	
North	67 (10.7)	35/67 (52.2)	32/67 (47.8)	
Toronto	36 (5.7)	13/36 (36.1)	23/36 (63.9)	
West	236 (37.6)	88/236 (37.3)	148/236 (62.7)	
Operator				
Charitable	57 (9.1)	18/57 (31.6)	39/57 (68.4)	.55
Municipal	101 (16.1)	42/101 (41.6)	59/101 (58.4)	
Nonprofit	117 (18.7)	47/117 (40.2)	70/117 (59.8)	
For profit	361 (57.6)	165/361 (45.7)	196/361 (54.3)	

Abbreviation: COVID-19, coronavirus disease 2019; NA, not applicable.

^a^ Cases include both suspected and laboratory-confirmed COVID-19 infections.

^b^ Differences in bed number tested with Wilcoxon rank sum test; differences in proportion tested by χ^2^ test.

Of 1 731 315 individuals older than 69 years living in Ontario during the study period, 229 (<0.1%) died; of 79 498 potential residents in LTC facilities, 83 (0.1%) died. The IRR for death due to COVID-19 was 13.1 (95% CI, 9.9-17.3) in the LTC population compared with community-living Ontario residents older than 69 years. When the whole community-living population was used as the referent, the IRR for death among the LTC population was 90.4 (95% CI, 68.9-117.6); when compared with community-living adults older than 59 years, 23.1 (95% CI, 17.6-30.2); and when compared with community-living adults aged 80 years and older, 7.6 (95% CI, 5.5-10.4) (Table 2).

**Table 2.** IRR for Coronavirus Disease 2019 Mortality in Long-term Care Residence^a^

Comparator population	Total deaths	Comparator population size	IRR (95% CI)
All ages	269	14 566 547	90.4 (68.9-117.6)
≥60 y	252	3 447 723	23.1 (17.6-30.2)
≥70 y	229	1 731 315	13.1 (9.9-17.3)
≥80 y	169	642 571	7.6 (5.5-10.4)

Abbreviation: IRR, incidence rate ratio.

^a^ Using mortality data reported to April 11, 2020. A total of 83 deaths were reported in long-term care facilities to April 7, 2020, among 79 498 potential residents.

**Figure 1.**
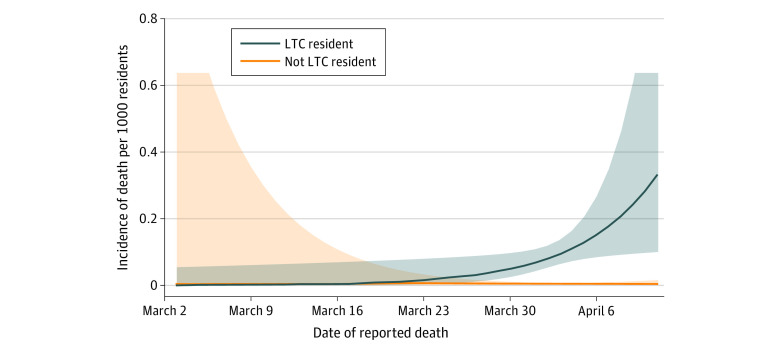
Model-Based Estimation of Coronavirus Disease 2019 Death Risk in Ontario The 2 curves represent modeled deaths per 1000 individuals in the long-term care (LTC) population and the community-dwelling population older than 69 years in Ontario. Shaded areas represent 95% CIs.

Projected trends in risk of COVID-19–related death among LTC residents were generated using model estimations that incorporated time × LTC resident status as an interaction term (Figure 1). The median IRR for COVID-19 death in LTC residents compared with community-dwelling individuals rose from 8.03 (95% CrI, 1.96-23.32) on March 29 to 87.28 (95% CrI, 6.44-769.76) by April 11, 2020.

**Figure 2.**
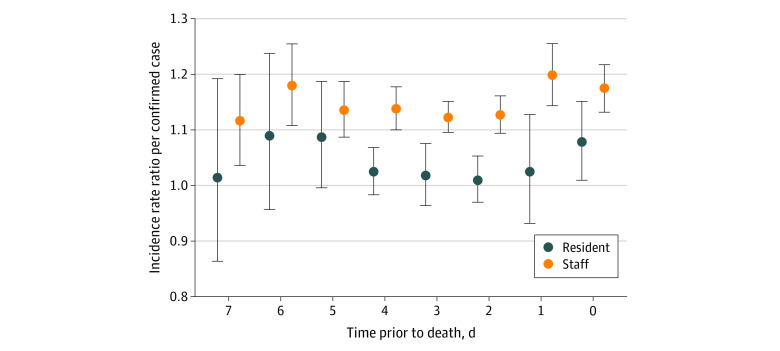
Incidence Rate Ratios for Death in Long-term Care, by Lagged Infections Among Residents and Staff Incidence rate ratios from Poisson regression models evaluating lagged associations between staff and residents with confirmed coronavirus disease 2019. Circles represent incidence rate ratios; vertical lines, 95% CIs. Elevated risk is seen with staff infections at all lags. Elevated risk is seen only with resident infections at a 0-day lag (ie, simultaneous with deaths).

In analyses focused on risk of death within LTC facilities, we found that lagged infections among staff were associated with death among residents (Figure 2) and were significant at all lags (0 to 7 days) after adjustment for date and numbers of residents with infection. The strongest associations were seen with staff with infection at a 2-day lag (relative increase in risk of death per staff member with infection, 1.20; 95% CI, 1.14-1.26) and a 6-day lag (relative increase in risk of death per staff member with infection, 1.17; 95% CI, 1.11-1.26). In contrast, the association between infection in residents and subsequent death was variable and far weaker than the associations seen for staff. It was statistically significant only at a 0-day lag (increased risk of death per infected resident, 1.08; 95% CI, 1.01-1.15).

## Discussion

In this analysis, we documented the rapid spread of COVID-19 through Ontario’s LTC system, with a marked increase in risk of death among older residents with frailty during a brief period from late March to early April 2020. Issues such as crowding, use of communal space, low staffing ratios, and high care needs (with resultant high density of physical contact between residents and staff) have long been recognized as key drivers of susceptibility to outbreaks in the LTC facility setting.^4,7^ In the context of COVID-19, this susceptibility has proven particularly deadly, with (as we demonstrate here) an incidence of mortality more than 13 times greater than that seen in community-living adults older than 69 years during a similar period, with relative risk of death rising sharply over time.

We also found that documented infection in facility staff, as opposed to residents, is a strong identifiable risk factor for mortality in residents, with temporality suggesting that residents are infected by staff and not vice versa. Although it might be argued that the limited nature of testing and the tendency to test staff and residents for COVID-19 after a resident dies might lead to spurious associations between identified infections and deaths, such spurious associations might result in equivalent effect sizes between residents and staff, rather than the divergent effect sizes observed here. The greater mobility and connectedness of staff, compared with residents, lends biological plausibility to this association.^11^

Transmission of infection is not the only mechanism by which infection in staff could result in increased mortality in an older population. Fear of COVID-19 could result in absenteeism by staff, which could itself lead to death through dehydration and other mechanisms in a high-needs population, which would be consistent with the lagged associations we observed here. Such tragedies have been documented in Canada recently.^12^ While this analysis was completed in April 2020, the subsequent course of events in Ontario has validated the trends we identified; as of June 10, 2020, 1766 COVID-19–related deaths have occurred among LTC facility residents in Ontario; deaths among residents of LTC facilities constituted 71% of all COVID-19 deaths in Ontario. Furthermore, 7 LTC facility staff and volunteers have died of the disease.^13^ Across Canada, it is estimated that more than 80% of COVID-19 deaths have occurred in the LTC facility setting; similar epidemiology is documented in the United States^14^ and several European countries,^15,16^ suggesting that our findings are likely to be generalizable.

The prevention of such deaths requires strategic guidance from health regions and the provision of sufficient testing and personal protective equipment. Provision of personal protective equipment has benefits both in bidirectional prevention of SARS-CoV-2 transmission and in providing workers peace of mind to stay on the job. Expanded testing, including testing of minimally symptomatic infection, will facilitate the early identification of infection and the implementation of effective infection control strategies. Integrated regional approaches to LTC facility human resource management, such as limiting workers to a single facility and ensuring that these workers earn a living wage to prevent the need for multiple jobs while at the same time maintaining adequate staffing levels, are also needed.^17^

### Limitations

Like any observational study, this study has limitations, including possible incompleteness of data collected rapidly during an outbreak, inconsistency in testing across Ontario, and absence of individual-level data on LTC facility infections and deaths. We have been unable to explicitly structure autocorrelation in our time series owing to effects on standard errors resulting from the limited size of our data set. We regard our outcome of interest, ie, death from COVID-19 among residents of LTC facilities, to be less likely misclassified than nonfatal infection among staff and residents. If misclassification of infection status in these individuals occurs at random, that would likely mean the associations reported here are lower-bound effect sizes. If underidentification of both fatal and nonfatal infections among residents and staff are clustered by home, that would result in association estimates that are biased upward. The temporality in the associations we observed provides a degree of reassurance in this regard.

## Conclusions

This study documented that the rapid movement of COVID-19 through Ontario’s LTC facility system has resulted in a marked surge in mortality in that population relative to community-living older adults. We found evidence that associates mortality with infection among LTC staff, highlighting the urgent need for improved infection control, more widespread testing, access to personal protective equipment, and economic protections and support for those who do this important work.
